# Delayed diagnosis of STAT1 gain-of-function variant in a patient with multiple endocrine autoimmunity and recurrent fungal infections

**DOI:** 10.1530/EDM-25-0054

**Published:** 2025-07-24

**Authors:** Sydney Sparanese, Rae Brager, David Fahmy, Jenny Garkaby

**Affiliations:** ^1^ Department of Medicine, University of British Columbia, Vancouver, Canada; ^2^ Department of Pediatrics, Division of Immunology, Allergy, and Dermatology, Hamilton Health Sciences, McMaster University, Hamilton, Canada; ^3^ Department of Medicine, Clinical Immunology and Allergy, Hamilton Health Sciences, McMaster University, Hamilton, Canada

**Keywords:** autoimmune, type 1 diabetes, hypothyroidism, immunodeficiency, case report

## Abstract

**Summary:**

This case report describes a 54-year-old woman with multiple endocrine autoimmune pathologies and recurrent mucocutaneous *Candida spp*. infections that were inappropriately attributed to her glycemic control. Following an allergic reaction over four decades later, the patient was referred to clinical immunology. The combination of persistent Candida infections, autoimmune endocrinopathies, and a positive family history prompted investigation for an inborn error of immunity (IEI). Genetic testing revealed a novel, missense mutation in STAT1. Functional analysis confirmed enhanced STAT1 protein phosphorylation, confirming a gain-of-function phenotype that explained her infectious and autoimmune manifestations. She was started on the JAK inhibitor, ruxolitinib, with clinical improvement. This case underscores the shared molecular mechanisms between IEIs and autoimmune endocrinopathies and highlights the importance of early recognition of IEI in patients with unusual or treatment-refractory infections alongside autoimmune disease. Endocrinologists and primary care providers may be the first to encounter such patients and should consider referral for immunologic and genetic evaluation. Early diagnosis can reduce long-term morbidity and open the door to targeted therapies that address the root cause of immune dysregulation.

**Learning points:**

- Persistent mucocutaneous candidiasis in patients with autoimmune endocrinopathies warrants evaluation for underlying IEI: while candidiasis is common in individuals with diabetes, recurrent or treatment-refractory infections – particularly in the presence of additional autoimmune conditions – should prompt consideration of IEI, including STAT1 gain-of-function mutations.
- Autoimmunity and immunodeficiency represent overlapping spectra of immune dysregulation: genetic syndromes such as STAT1 GOF may manifest with both autoimmune endocrinopathies and increased susceptibility to fungal infections, underscoring the importance of a unifying diagnostic approach to seemingly disparate clinical features.
- Early referral to clinical immunology and genetic testing can enable timely diagnosis and targeted therapy: early recognition of IEI allows for disease-modifying treatment, such as JAK inhibition, which may alleviate infectious susceptibility and autoimmune manifestations, ultimately reducing morbidity and improving the quality of life.
- A thorough family history can provide critical diagnostic clues in cases of immune dysregulation: subtle patterns of autoimmunity or recurrent infections in family members – particularly in non-consanguineous pedigrees – may indicate heritable immunologic disorders and should inform the clinical threshold for pursuing genetic evaluation.

## Background

Autoimmune conditions are characterized by a dysregulated immune response, which leads to immune-mediated targeting of healthy tissue. Autoimmune endocrinopathies such as type 1 diabetes mellitus (T1DM) and hypothyroidism are the most common manifestations of autoimmunity (1, 2). There is considerable overlap in the genetic causes of autoimmune diseases and inborn errors of immunity (IEI). Autoimmune or autoinflammatory complications are observed in more than 25% of patients with IEI (3). The diverse manifestations of immune dysregulation and lack of recognition of rare syndromes as such can lead to diagnostic challenges and delays. For patients, this translates into increased complications, worse prognosis, prolonged suffering and early mortality, underscoring the importance of early recognition of the relationship between IEI and autoimmune symptoms.

Hyperglycemia, among other proposed mechanisms, renders diabetic patients more susceptible to opportunistic infections, including fungal infections. Chronic mucocutaneous candidiasis (CMCC) is characterized by persistent or recurrent *Candida* spp. infections of the nails, skin, scalp, oral and/or genital mucose. CMCC is an AIDS-defining illness in patients with HIV but can also be a harbinger of IEI, particularly T-cell defects. Specifically, T-helper 17 (Th17) cells play a central role in defending against extracellular pathogens such as fungi through cytokine production and neutrophil recruitment. Unsurprisingly, IEI that involve impaired Th17 immunity result in susceptibility to recurrent and severe fungal infections. One such genetic syndrome is STAT1 gain-of-function (GOF), a heterogeneous IEI that underlies autosomal dominant CMCC.

Here, we report the case of a patient with T1DM and Hashimoto’s thyroiditis presenting with recurrent nail, oropharyngeal and esophageal candidiasis who was referred to an allergy specialist for an allergic reaction to antifungal medication. This consultation led to the diagnosis of a novel, heterozygous pathogenic change in *STAT1* and the resulting diagnosis of STAT1 GOF.

## Case presentation

The patient is a 54-year-old female with a history of T1DM, diagnosed at age 14, and Hashimoto’s thyroiditis, diagnosed at age 20. Despite adequate glycemic control, she suffered from recurrent CMCC starting at age 16 that required nearly monthly use of oral antifungal treatment for resolution. Multiple tongue scrapings had been positive for *Candida albicans.* As a young adult, she had recurrent vaginal candidiasis. At age 36, dysphagia and odynophagia prompted referral to gastroenterology for upper endoscopy, which demonstrated esophageal candidiasis and stricturing disease necessitating subsequent dilation. She had one episode of paronychia requiring intravenous antifungal medication and onychomycoses of multiple nails. She denied any substance use. She is the only child of non-consanguineous parents of European descent. Family history was remarkable for Hashimoto’s thyroiditis and recurrent fungal nail infections in patient’s mother before her death at age 74. No cause of death was elucidated on autopsy.

Due to the recurrent nature of her fungal infections, she had been educated that the most likely cause was poorly controlled DM and resultant hyperglycemia, for which she was referred to a dietitian. Despite self-reported adequate glycemic control, the fungal infections persisted.

## Investigation

At the age of 48, she developed urticaria after receiving fluconazole and was referred to allergy and clinical immunology for suspected drug allergy. However, the constellation of CMCC, T1DM and autoimmune hypothyroidism prompted consideration of IEI, and a workup was initiated (Table 1). The patient underwent a genetic evaluation with a clinical IEI panel which demonstrated a novel heterozygous variant in *STAT1* c.842A>G (p.E281G). This variant was absent from large population databases (gnomAD v4.0) and was classified as a variant of uncertain significance. Given the strong concordance between the clinical phenotype and the genetic findings, this monogenic variant was suspected to underlie the patient’s symptoms. To confirm a diagnosis of STAT1 GOF, a functional assessment of STAT1 phosphorylation was performed. Peripheral blood mononuclear cells (PBMCs) from the patient and healthy controls were stimulated with recombinant IL-6 for 15 min (Jeffrey Modell Diagnostic and Research Center for Primary Immunodeficiency at the Medical College of Wisconsin). Phosphorylated STAT1 (pSTAT1) was assessed by flow cytometry at 15, 30, 60, and 120 min post-stimulation. The patient’s PBMCs exhibited elevated pSTAT1 levels at all measured time points, with delayed dephosphorylation when compared to healthy controls, confirming a GOF phenotype.

**Table 1:** Results of initial laboratory investigations. Abnormal values are in bold.

Test	Value	Reference range
WBC (×10^9^/L)	4.1	4.0–11
Hemoglobin (g/L)	**100**	115–165
Platelets (×10^9^/L)	240	150–400
Neutrophils (×10^9^/L)	2.2	2.0–7.5
Lymphocytes (×10^9^/L)	**1.4**	1.5–4.0
Eosinophils (×10^9^/L)	0.1	0.0–0.4
Monocytes (×10^9^/L)	0.4	0.2–0.8
Basophils (×10^9^/L)	0.1	0.0–0.1
CD3+ Absolute cell count (×10^9^/L)	**0.69**	1.0–3.6
CD3+/CD4+ Absolute cell count (×10^9^/L)	**0.46**	0.5–1.8
CD3+/CD8+ Absolute cell count (×10^9^/L)	0.20	0.2–0.8
CD19+ Absolute cell count (×10^9^/L)	0.34	0.1–0.8
NK %	13	5–26
PHA stimulation index	4.13	≥1.4
IgG (g/L)	14.4	6.4–14.7
IgM (g/L)	0.99	0.4–2.1
IgA (g/L)	3.1	0.7–3.5
IgE (KIU/mL)	3	<87
Anti-tetanus Ab (IU/mL)	4.75	>0.1
Anti – measles-, mumps-, and rubella-specific IgG	Reactive to all	
Varicella-specific IgG	Non-reactive	
TSH (mIU/L)	0.70	0.73–4.34
Free T4 (pmol/L)	26.2	10.8–28.2
HbA1c (%)	**7.7**	4.0–6.0

## Treatment

Her diagnosis led to initiation of a targeted therapy, Ruxolitinib, an inhibitor of Janus kinase (JAK) 1 and 2, known to be efficacious in decreasing STAT1 GOF symptoms, including CMCC (4). Ruxolitinib was initiated at a dose of 5 mg twice daily, with the plan to titrate to effect over time.

## Outcome and follow-up

In the 5 months since initiating therapy, she has had steady improvement in lymphocyte counts and interval improvement of her CMCC.

## Discussion

Endocrine organs are common targets of autoimmune manifestations within the spectrum of IEI, typically resulting in hypofunction. The shared genetic and molecular pathways underlying these disorders underscore the role of immune tolerance and regulatory mechanisms in maintaining endocrine homeostasis. For example, T1DM occurs in up to 18% of patients with autoimmune polyendocrinopathy-candidiasis-ectodermal dystrophy (APECED), caused by functional defects in *AIRE*, a gene responsible for assessing the self-avidity of developing T-cells in the thymus. More than half of these patients have multiple endocrine deficiencies (5). Another such syndrome is IPEX (immune dysregulation, polyendocrinopathy, enteropathy, X-linked), a rare IEI caused by pathogenic changes in *FOXP3,* a transcription factor critical for the development and function of regulatory T cells (Tregs). The subsequent disruption in immune tolerance leads to widespread autoimmune destruction, particularly in the endocrine and gastrointestinal systems. Other genetic defects that lead to endocrinopathies include loss-of-function changes in *IL2RA, STAT5B* and GOF in *STAT3* and *STAT1*. Common variable immunodeficiency (CVID) results in hypogammaglobulinemia, impaired antibody production and increased susceptibility to infections. Between 20 and 30% of patients with common variable immunodeficiency (CVID) will have or go on to develop autoimmune symptoms, including endocrinopathies (6). In addition, a high prevalence of anterior pituitary (secondary hypogonadism and hypothyroidism) and primary endocrine dysfunction is observed in adults with antibody deficiencies, including CVID. In the largest cohort of patients with STAT1 GOF, autoimmune diagnoses were present in 37% of patients, with endocrinopathy being the most common manifestation (7). Table 2 summarizes a subset of immunodeficiency syndromes with autoimmune endocrine overlap. These diseases highlight the shared genetic underpinnings of autoimmunity and immunodeficiency.

**Table 2:** Red flag symptoms of primary immunodeficiencies with overlapping endocrine autoimmunity.

Disease	Pathophysiology	Red flag symptoms	Most common endocrinopathies
IPEX syndrome and IPEX-like disorders	Systemic autoimmune disorder caused by mutations in *FOXP3*, *CTLA4*, *LRBA, IL2RA* and other genes, leading to Treg dysfunction	Severe enteropathy Atopic dermatitis Autoimmune cytopenias Hepatitis	T1DM Autoimmune hypothyroidism
APECED	Autosomal recessive disorder caused by mutations in *AIRE*, leading to impaired thymic-negative selection of self-reactive T-cells – resulting in multi-organ autoimmunity and susceptibility to specific infections	Chronic mucocutaneous candidiasis Ectodermal dystrophy – leading to dental enamel hypoplasia and susceptibility to dental carries, nail dystrophy, keratopathy and corneal abnormalities, alopecia	Hypoparathyroidism Adrenal insufficiency
CVID	Heterogeneous group of disorders characterized by impaired B-cell differentiation and antibody production, leading to recurrent infections and immune dysregulation	Recurrent sinopulmonary infections (particularly with encapsulated bacteria) Bronchiectasis Autoimmune cytopenias Enteropathy, chronic diarrhea, malabsorption and weight loss Lymphoid malignancies	T1DM Autoimmune hypothyroidism
*STAT3* GOF	Autosomal dominant disorder characterized by mutations in *STAT3,* leading to aberrant Treg and Th17 cell function and differentiation	Most present with severe, early onset or multi-system autoimmunity involving endocrine, hematologic, gastrointestinal systems Lymphoproliferative disorders Short stature	Autoimmune hypothyroidism T1DM Growth hormone deficiency
*STAT1* GOF	Autosomal dominant disorder characterized by mutations in *STAT1,* leading to impaired Th17 cell development and subsequent loss of immune tolerance, and impaired mucosal immunity	Persistent, severe or recurrent *Candida spp.* infections Most develop autoimmune manifestations by third decade of life – primary endocrine and/or hematologic Increased risk of cerebral aneurysms	T1DM Autoimmune hypothyroidism

IPEX, immune dysregulation, polyendocrinopathy, enteropathy, X-linked syndrome; Treg, regulatory T cell; APECED, autoimmune polyendocrinopathy-candidiasis-ectodermal dystrophy; CVID, common variable immunodeficiency; Th17, T helper 17; T1DM, type 1 diabetes mellitus.

While the sequelae of hyperglycemia can drive *Candida spp.* infections, this case emphasizes the need for clinicians to maintain clinical suspicion for IEI and initiate early referral to clinical immunology. The severity and persistence of infections despite optimal glycemic control, coupled with a suspicious family history, are highly suggestive of an IEI. In APECED, the initial manifestation of disease in most patients is oral candidiasis, typically predating the onset of endocrine deficiencies (5). We aim to increase the awareness of the overlapping features between IEI and autoimmune endocrinopathies among endocrinologists as they are frequently the initial points of contact with the medical system. Clues that may suggest a genetic cause for immune dysregulation include the early onset of autoimmunity, multiple autoimmune diagnoses, and a family history of an autoimmune process.

While wider application of genetic analysis has improved the accessibility to an IEI diagnosis, the continued emergence of new genetic mutations requires functional validation to confirm a definitive diagnosis. More than 75 individual mutations have been reported to cause STAT1 GOF through hyperphosphorylation of STAT1 (pSTAT1) in immune cells, either at baseline or following cytokine stimulation. As such, the current standard of practice for validating STAT1 mutations that confer GOF relies on comparing pSTAT1 levels between a potential index case and healthy controls. Functional analysis of our patient performed in a clinical diagnostic laboratory revealed increased pSTAT1 in comparison to healthy controls, confirming the diagnosis of a GOF phenotype. Based on our data and the 2015 American College of Medical Genetics (ACMG) guideline, this novel variant in STAT1 can now be classified as pathogenic as it satisfies the PS3, PM6, PP2, and PP4 criteria.

Recognition of these patients is critical not only to pursue an accurate diagnosis, but also to facilitate targeted medical therapy that addresses both immune dysfunction and endocrinopathies. JAK inhibitors such as ruxolitinib have been shown to reduce STAT1 hyperphosphorylation in STAT1 GOF patients, ameliorating not only the CMCC but also symptoms of autoimmunity (8). Comparatively, conventional therapy for CMCC includes the use of systemic and/or topical antifungals for treatment and prophylaxis, leading to progressive antimicrobial resistance, and rendering this as an ineffective long-term strategy. The consequences of CMCC are significant; in addition to the day-to-day morbidity, it is associated with an increased risk of esophageal cancer (9). Moreover, targeted therapy in IEI has been shown to reverse autoimmunity. A 2020 case report described a 17-year-old patient with T1DM and CMCC who was found to have a heterozygous STAT1 GOF mutation. Twelve months after initiation of ruxolitinib, the patient was able to completely discontinue insulin therapy and remained euglycemic for another year thereafter (10). The importance of genetic testing to identify a diagnosis cannot be overstated given the potential for a remarkable response to targeted therapy.

Our case presents an adult with early onset endocrine autoimmunity, recalcitrant CMCC, and a family history of similar symptoms. A suspected drug allergy prompted referral to Allergy and Clinical Immunology, which ultimately led to the diagnosis of a novel STAT1 GOF change, four decades after her initial presentation. We suspect that the STAT1 change was maternally inherited and expressed in an autosomal dominant pattern given the proband’s mother’s history of autoimmunity and possible onychomycosis. Despite the diagnostic delay for this patient, there has been marked symptomatic improvement since the initiation of targeted therapy for STAT1 GOF.

## Conclusion

The diagnosis of IEI is often delayed due to the diverse manifestations of disease, heterogeneity in presentations, and lack of awareness of rare disorders. Careful consideration for a genetic underpinning of disease should be given to patients with unexplained, opportunistic infections, multiple autoimmune manifestations, or a positive family history. This holds particularly true for clinicians who may be the first point of contact for the treatment of an autoimmune condition, such as T1DM or autoimmune thyroid disease. This case highlights the need for higher clinical suspicion of IEI among patients with autoimmune endocrine disease and underscores the importance of early referral for assessment by a clinical immunologist. As targeted therapies become more widely available, genetic diagnosis opens the door to options to treat and potentially reverse these autoimmune and infectious conditions, allowing patients to avoid unnecessary morbidity and mortality.

## Declaration of interest

The authors declare that there is no competing conflict of interest that could be perceived as prejudicing the impartiality of the work reported.

## Funding

This work did not receive any specific grant from any funding agency in the public, commercial or not-for-profit sector.

## Patient consent

Written informed consent for the publication of their clinical details was obtained from the patient.

## Author contribution statement

SS drafted and contributed to manuscript production. RB, DF, and JG analyzed and interpreted the patient data and contributed to the care of the patient. JG conceptualized the case report. All authors read and approved the final manuscript.
